# Recurrence Risk of Renal Cell Carcinoma Lingers Even Decades After Nephrectomy

**DOI:** 10.7759/cureus.17217

**Published:** 2021-08-16

**Authors:** Muhammad Ammar B Hamid, Aasim Sehbai, Shahan Tariq, Sana Ullah

**Affiliations:** 1 Hematology and Oncology, Alabama Cancer Care (ALCC), Anniston, USA; 2 Oncology, Atomic Energy Cancer Hospital (NORI), Islamabad, PAK

**Keywords:** renal cell carcinoma, renal cell metastasis, metastasis, lung nodule, renal neoplasm

## Abstract

Renal cell carcinoma (RCC) is a common malignancy in elderly males. Metastatic spread of this cancer is not an uncommon occurrence, even after nephrectomy. Lung, bone, liver, and brain are the most frequently involved sites. Such a type of presentation mostly occurs within five years after nephrectomy however, cases have been reported later as well. Here, we report a case of metastatic renal cell carcinoma that presented in the form of a lung growth 28 years after nephrectomy. This highlights the importance to consider relapsed metastatic renal cancer in the differential, even decades after its surgical removal.

## Introduction

Renal cell carcinoma (RCC) usually originates from the cortex of the kidney. It is mostly seen in men between 50-70 years of age. The major risk factors that contribute to disease are raised blood pressure, high body mass index, older age, and polycystic kidney disease [1]. The most common sites for the metastases of RCC include the lung, bone, liver, and brain. Although infrequent, yet the metastatic presentation of such lesions more than five years after nephrectomy is not an uncommon finding [2]. In this case report, we describe the unique presentation of a metastatic lung nodule in a patient 28 years after remaining in remission post nephrectomy.

## Case presentation

An 82-year-old man with a history of right-sided renal cell carcinoma underwent nephrectomy in 1991 and had been in remission for 28 years. About a year ago, he started to develop respiratory symptoms such as cough and shortness of breath over the course of two to three weeks. Initially, an infective etiology was suspected and the patient was advised antibiotics and bronchodilators. In addition to consolidation, the chest x-ray also detected a nodular density in the left lung. Hence, the patient was scheduled for a positron emission tomography/computed tomography (PET/CT) scan to further characterize the lesion.

**Figure 1:**
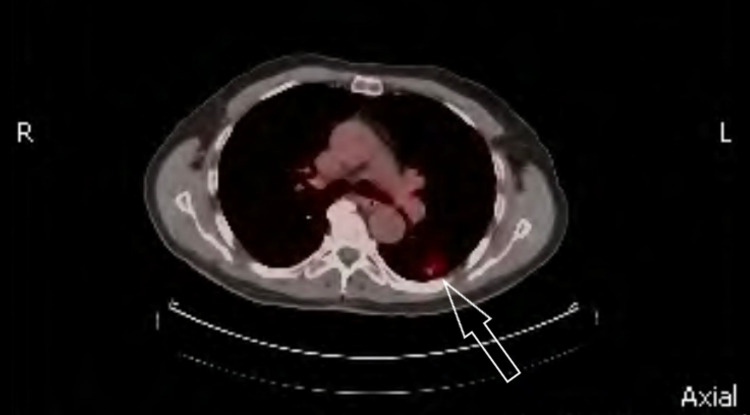
A PET/CT scan demonstrating a nodule in the posterior aspect of the left lower lobe of the lung

**Figure 2:**
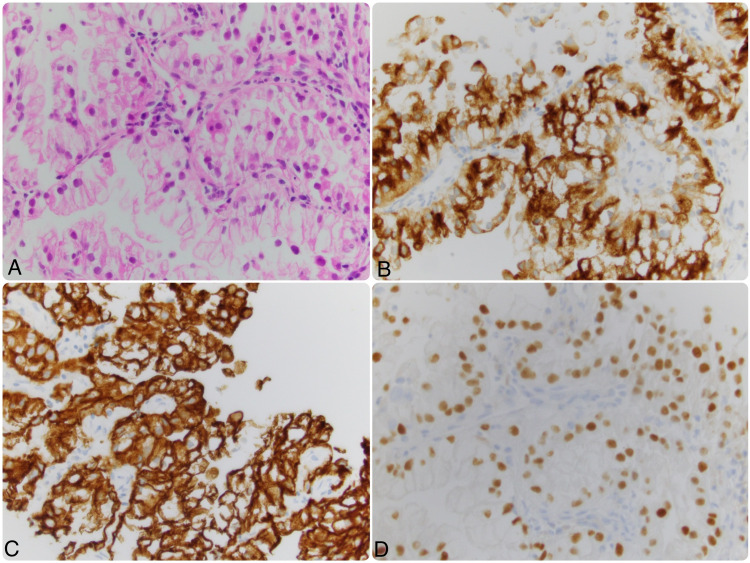
Histopathological/Immuno-histochemical evaluation of the biopsied tissue A) Microscopic examination demonstrating nest of epithelial cells with clear cytoplasm (H&E 40x). B) Immuno-histochemistry positive for RCC stain. C) Pan-cytokeratin stain positive. D) PAX-8 stain positive.

During this period, he developed acute shortness of breath and underwent bronchoscopy. A mucus plug was removed from the right bronchus however, no endo-bronchial lesion could be appreciated. A PET/CT scan revealed a well-circumscribed 1.6 x 1.0 cm pulmonary nodule with a standardized uptake value (SUV) of 3.4 in the posterior aspect of the left lower lobe, mild lymphadenopathy in the right hilum, and benign-appearing cysts in the liver/spleen (Figure 1). MRI of the brain was negative. A diagnostic dilemma emerged as there was uncertainty regarding its true nature i.e either a primary growth or a relapsed version of metastatic RCC. Therefore, a CT-guided biopsy was performed and pathology was consistent with metastatic renal cell carcinoma. Immunochemical staining was positive for pan-cytokeratin, RCC stain, and paired box gene 8 (PAX-8) whereas, P63 staining was negative (Figure 2). Thus, a diagnosis of relapsed metastatic RCC was confirmed and its presentation after 28 years was indeed thought-provoking.

Family history was remarkable for colon cancer in his mother and lung cancer in his brother. He was a non-smoker and reported an allergic reaction to meperidine. Past medical history was significant for cataracts, osteoarthritis, and peptic ulcer disease.

The case was discussed on the tumor board and the best line of approach was decided as systemic immunotherapy with nivolumab and ipilimumab every three weeks for six cycles. The goal of this therapy was palliative and symptom control. Re-imaging after six cycles revealed good results with no suspicious hypermetabolic foci. Nonspecific mild nodularity in the left lower lobe was observed which was later targeted with radiation. He was monitored on a regular basis.

Imaging on subsequent follow-up was suspicious due to increased anteroposterior window node. Thus, endoscopic ultrasound with biopsy was advised. A single 23 x 16 mm malignant appearing lymph node in the superior mediastinum was found. Fine needle aspiration was positive for malignant RCC cells. After a detailed discussion of all risks and benefits with the patient, immunotherapy was deemed as the most suitable option. Due to his excellent response earlier, nivolumab is being re-administered. Treatment with axitinib might also be considered in the future depending on disease progression.

## Discussion

Over the years, a significant amount of literature has been gathered on patients having late recurrences of RCC where unusual metastatic sites were involved; scalp, submandibular glands, thyroid, and pancreas to name a few. Although the more common sites of metastasis are lungs, liver, and bone. Nonetheless, the risk of recurrence was considerably higher within the first five years [3]. The exact mechanism leading to differences in the sites of metastasis still remains unknown [3]. Exploring the diagnostic variables along with the analysis of underlying pathophysiology paves the path for optimization of treatment, as well as provides an approximation regarding treatment effectiveness and future prognosis. Despite the fact that there are currently no markers/diagnostic tests available that can help precisely predict the exact time/location of metastasis, a general idea of how likely it is to recur can be gauged looking at researches done over the past several years.

A number of clinical trials suggest that the spread of metastatic tumor cells from RCC most commonly occurs in the lungs [4]. Such lesions usually lead to symptoms like pleuritic chest pain, shortness of breath, cough, and even hemoptysis. However, there is a probability of finding these lesions in patients who are asymptomatic incidentally via imaging [5]. CT scans of the chest seem to be more sensitive in detecting lung metastases [6].

On the other hand, there are several limitations associated with the modified/generalizable protocols of the American Urological Association (AUA) and the National Comprehensive Cancer Network (NCCN). Even after rigorously adhering to the guidelines, research has indicated that recurrences of RCC continue to remain unnoticed until later stages; around 33%. Moreover, RCC recurrences do happen after the five-year period of mandatory surveillance window [7].

Retrospective trials have a bias owing to the selection of patients augmented by the lack of a comparator body. A prospective and randomized controlled analysis using blinds for surveillance techniques would help to get rid of such biases. It should be noted though, that recurrences diagnosed relatively earlier, using increased duration and frequency of imaging, almost always show an apparent gain in survival, owing to lead time bias [8]. This implies developing more sensitive, accurate, and overall cost-effective post-op surveillance as well as strategies for long-term screening follow-ups, not to mention effective treatment options to improve the overall survival of patients.

Gene profiling may lead the way to a more precise estimation of risk factors in patients with RCC in the future [9]. It will eventually result in more efficient use of surveillance regimens which shall ultimately prioritize those patients in whom survival benefit has the most weightage. Furthermore, it shall also help to optimize interventions resulting in better patient outcomes.

## Conclusions

A tool for the stratification of patients with a higher risk of renal cell carcinoma metastasis/recurrence is the need of the hour. This shall help bypass the limitations that currently exist in the protocols, allowing patients to be diagnosed much earlier and quantify recurrences on the earliest signs of biochemical relapse. The development of newer treatment modalities that provide a mortality benefit and may even prevent late recurrences are indeed necessary to achieve an optimum personalized management goal in this heterogeneous disease.
